# Genome Sequence of the Euphotic Hawaiian Gammaproteobacterium HetDA_MAG_MS8, in the Order *Nevskiales*, Family *Oceanococcaceae*, Genus *Oceanococcus*

**DOI:** 10.1128/mra.00592-22

**Published:** 2022-12-14

**Authors:** Kaveh A. Mahdavi, Elaina D. Graham, John F. Heidelberg, Eric A. Webb

**Affiliations:** a Department of Biological Sciences, University of Southern California, Los Angeles, California, USA; b Department of Biological Sciences, Marine Environmental Biology Section, University of Southern California, Los Angeles, California, USA; Montana State University

## Abstract

We present a metagenome-assembled genome (MAG), HetDA_MAG_MS8, that was determined to be unique via relative evolutionary divergence (RED) scores and average nucleotide identity (ANI) values. HetDA_MAG_MS8 is in the order *Nevskiales*, genus *Oceanococcus*, and was assembled from a heterocytous cyanobiont enrichment from the Hawaii Ocean Time Series. HetDA_MAG_MS8 is predicted to be a facultative, aerobic, anoxygenic photolithoheterotroph that has the potential for sulfide oxidation and dimethylsulfoniopropionate (DMSP) synthesis.

## ANNOUNCEMENT

Here, we describe a metagenome-assembled genome (MAG) that was isolated from a heterocytous diazotroph enrichment, HetDA_MAG_MS3, and was originally observed growing in *Trichodesmium* colonies by Momper et al. (1). Momper et al. (1) found a heterocystous diazotrophic cyanobacterium (HetDA) living in association with *Trichodesmium* spp. Briefly, *Trichodesmium* spp. were collected from the top 10 m with a 130-μm plankton net, and colonies were hand-picked (1) and incubated in sterile YBC-II medium at 24°C with a 12-h light (100 μmol photons m^−2^ s^−1^)/12-h dark cycle. The enrichment was grown under the aforementioned conditions for 5 years prior to sequencing and was transferred to fresh medium every month. DNA was extracted from a 50-mL subsample of the enrichment using a Qiagen DNeasy PowerSoil kit according to the manufacturer's instructions. The DNA was sent to the University of Southern California Epigenome Center and sequenced on an Illumina MiSeq sequencer (paired-end 250-bp reads, with a total of 4,725,335 raw reads) using the MiSeq reagent kit v2 with 300 cycles. Reads were trimmed using Trimmomatic v0.38 (parameters: –phred33, ILLUMINACLIP:TruSeq3-PE.fa:2:30:10 SLIDINGWINDOW:10:28 MINLEN:50) (2). Raw reads were assembled using MetaSPAdes v3.14.0 (3), mapped to the resulting assembly using Bowtie2 v2.3.5 (4), and filtered using CoverM v0.4.0 (parameters: –min-read-percent-identity 0.95 –min-read-aligned-percent 0.75) to calculate coverage (5). The resulting assembly was binned using MetaBat2 v2.12.1 (6), BinSanity-wf v0.3.8 (7), and Concoct v1.1.0 (8) with default parameters, and DASTool v1.1.2 (9) was used to determine a final set of MAGs. All bins were run through MetaSanity v1.3.0 (10), PhyloSanity, and FuncSanity for annotations using the default configuration file.

HetDA_MAG_MS8 was found to be unique via GTDB-tk (11) relative evolutionary divergence (RED) scores and average nucleotide identity (ANI) values within the *Gammaproteobacteria* order *Nevskiales* and the genus *Oceanococcus*. The assembled genome has 21 contigs, with an *N*_50_ value of 591,228 bp and a total size of 3,532,557 bp, and is 98.81% complete, as determined by CheckM (12). The genome has a GC content of 58.5%, a coding density of 90.98%, and 3,140 predicted genes. The closest relative of HetDA_MAG_MS8, with an ANI value of 76.82%, is Oceanococcus atlanticus, an aerobic heterotroph that was isolated from deep Atlantic sediment in an oil-degrading enrichment (13, 14). Three of the closest relatives of HetDA_MAG_MS8, namely, Oceanococcus atlanticus, Algiphulus aromaticivorans, and Polycyclovorans algicola, are capable of degrading pollutants; Oceanococcus atlanticus can degrade oil, hexadecane, and octacosane, and the other two species can degrade two- and three-ring polycyclic aromatic hydrocarbons (15).

Gene annotation and metabolic analysis suggest that HetDA_MAG_MS8 is a facultative aerobic anoxygenic photolithoheterotroph. It is suspected to metabolize organic carbon aerobically by using the tricarboxylic acid (TCA) cycle but appears to have the potential for metabolism under microoxygenic or anoxygenic conditions, because genes encoding proteins for glycolysis and mixed acid fermentation (ethanol and acetate to acetaldehyde), *cbb*_3_-type cytochrome *c* oxidase, and cytochrome *bd* complex proteins were found. Pathways to use sulfur as the electron source and an anoxygenic type 2 photosynthetic reaction center were detected but ribulose-1,5-bisphosphate carboxylase-oxygenase (RuBisCo) was not. DsyB is the key methyltransferase enzyme in the dimethylsulfoniopropionate (DMSP) synthesis pathway (16, 17). HetDA_MAG_MS8 also contains *dsyB*, which forms DMSP, the precursor to atmospheric dimethyl sulfide (DMS) which plays a significant role in the sulfur cycle, cloud formation, and climate regulation (18).

HetDA_MAG_MS8 appears to be an *Oceanococcus* sp. strain with the potential for aerobic anoxygenic photolithoheterotrophy.

### Data availability.

Raw sequences and MAGs are available under BioProject accession number PRJNA719568. Raw reads are available in the SRA under accession number SRR14140256. The genome is available under BioSample accession number SAMN18613315.
